# Dose–Response Relationships and Comparative Efficacy of Aldosterone Synthase Inhibitors in Resistant Hypertension: A Comprehensive Network Meta-Analysis and Meta-Regression

**DOI:** 10.3390/jcm15124477

**Published:** 2026-06-09

**Authors:** Kareem A. Mohamed, Mohamed Nasser Elshabrawi, Hossam Albeyoumi Mohammed, Mohamed Khalil, Ahmed AlGhazawy, Youssof Eshac, Atef Akoum, Ibrahim Kamal, Yasar Sattar, Ashesh Das, Mohammed Rahouma, Akshay Kumar

**Affiliations:** 1 Department of Internal Medicine, Hartford Hospital, Hartford, CT 06106, USA; kmohamed.md@gmail.com; 2 Clinical Research Department, Aswan Heart Centre, Magdi Yacoub Foundation, Aswan 81512, Egypt; mohamedshabrawi86@gmail.com; 3 Department of Internal Medicine, Uconn Health, Farmington, CT 06030, USA; albeyoumimohammed@uchc.edu (H.A.M.); mokhalil@uchc.edu (M.K.); alghazawy@uchc.edu (A.A.);; 4 Hennepin Healthcare, Minneapolis, MN 55404, USA; atef.akoum@hcmed.org; 5 Faculty of Medicine, Al-Azhar University, Cairo 11754, Egypt; ibrahem.kamoo98@gmail.com; 6 Department of Interventional Cardiology, West Virginia University, Morgantown, WV 26506, USA; mdyasarsattar@gmail.com; 7 Mayo Clinic, Rochester, MN 55905, USA; das.ashesh@mayo.edu; 8 Department of Cardiothoracic Surgery, Weill Cornell Medicine, NYU Langone Health 530, 1st Ave Suite 7R, New York, NY 10065, USA; mmr2011@med.corell.edu

**Keywords:** hypertension, aldosterone synthase inhibitors, renin–angiotensin system

## Abstract

**Background:** Resistant hypertension represents a significant clinical challenge, often driven by dysregulated aldosterone production. Aldosterone synthase inhibitors (ASIs) are a novel therapeutic class designed to directly suppress aldosterone biosynthesis. This study aimed to synthesize evidence regarding the comparative efficacy, optimal dosing, and dose–response relationships of three ASIs—baxdrostat, lorundrostat, and osilodrostat—for the treatment of resistant hypertension. **Methods:** A systematic review and network meta-analysis (NMA) were conducted following PRISMA 2020 guidelines. We searched PubMed, Web of Science, and CENTRAL for randomized controlled trials (RCTs) published up to 31 October 2025. The primary outcome was the mean change in systolic blood pressure (SBP) compared to placebo. We utilized a frequentist random-effects model, characterized dose–response relationships via meta-regression, and ranked treatments using the Surface Under the Cumulative Ranking Curve (SUCRA). **Results:** The analysis included 8 RCTs comprising 3253 participants. Baxdrostat 2 mg once daily demonstrated the greatest antihypertensive efficacy with a mean SBP reduction of −13.8 mmHg (95% CI: −17.5 to −10.1) versus placebo, followed by Lorundrostat 100 mg (−12.5 mmHg) and Baxdrostat 1 mg (−11.5 mmHg). SUCRA analysis identified Baxdrostat as the highest-ranked treatment (83.0%), followed by Lorundrostat (67.8%) and Osilodrostat (55.5%). Dose–response meta-regression revealed strong linear correlations for Baxdrostat (R^2^ = 0.91), Osilodrostat (R^2^ = 0.81), and Lorundrostat (R^2^ = 0.80). **Conclusions:** ASIs, particularly Baxdrostat and Lorundrostat, offer robust blood pressure reductions in patients with resistant hypertension. The strong linear dose–response relationships suggest these agents have a favorable therapeutic window for titration. While effective, clinical implementation requires careful monitoring for adverse events.

## 1. Introduction

Hypertension remains the leading modifiable risk factor for cardiovascular disease, stroke, and premature mortality worldwide, affecting approximately 1.28 billion adults globally and contributing to an estimated 10.8 million deaths annually [1,2]. Despite the availability of multiple antihypertensive drug classes, blood pressure (BP) control rates remain suboptimal, with only 43% of treated hypertensive patients achieving target BP levels in high-income countries and considerably lower rates in low- and middle-income nations [3,4]. This therapeutic gap is particularly pronounced in resistant hypertension (RH), defined as BP above goal despite concurrent use of three or more antihypertensive agents of different classes, ideally including a diuretic, or controlled BP requiring four or more medications [5]. Resistant hypertension affects 10–15% of the general hypertensive population and up to 30% of patients in specialized hypertension clinics, representing a substantial clinical challenge with significantly elevated cardiovascular risk [6].

The pathophysiology of resistant hypertension is multifactorial, but accumulating evidence implicates dysregulated aldosterone production and mineralocorticoid receptor (MR) activation as central mechanisms [7]. Aldosterone, the principal mineralocorticoid hormone synthesized by the adrenal zona glomerulosa, promotes sodium retention and potassium excretion. Beyond its renal effects, excess aldosterone exerts profound systemic consequences by driving myocardial and perivascular fibrosis, stimulating oxidative stress, and inducing endothelial dysfunction. Consequently, the systemic inhibition of aldosterone pathways is critical not only for blood pressure reduction but also for mitigating target-organ damage and adverse cardiovascular remodeling [8]. Notably, 10–20% of patients with resistant hypertension demonstrate primary aldosteronism, while many others exhibit inappropriate aldosterone secretion relative to sodium status [9]. Furthermore, aldosterone breakthrough—the phenomenon of rising aldosterone levels despite renin–angiotensin system (RAS) blockade—occurs in up to 40% of patients treated with angiotensin-converting enzyme inhibitors or angiotensin receptor blockers, potentially limiting the efficacy of these widely prescribed agents [10].

Additionally, pseudoresistance (e.g., medication non-adherence and white-coat effect) and lifestyle contributors such as obesity and high sodium intake complicate management. Secondary causes are particularly prevalent in this population, with obstructive sleep apnea (OSA) and primary aldosteronism (PA) being the most common, affecting approximately 20–30% and 10–20% of patients with resistant hypertension, respectively. Other less frequent secondary causes include renovascular disease, Cushing’s syndrome, and pheochromocytoma [11].

True resistant hypertension must be distinguished from pseudoresistance through a structured diagnostic evaluation, ideally utilizing 24 h ambulatory blood pressure monitoring (ABPM) to rule out white-coat effect and assessing medication adherence. Furthermore, clinical guidelines mandate screening for primary aldosteronism in this cohort using the plasma aldosterone-to-renin ratio (ARR), followed by confirmatory testing such as oral sodium loading or saline infusion, to guide targeted therapy.

**Figure 1:**
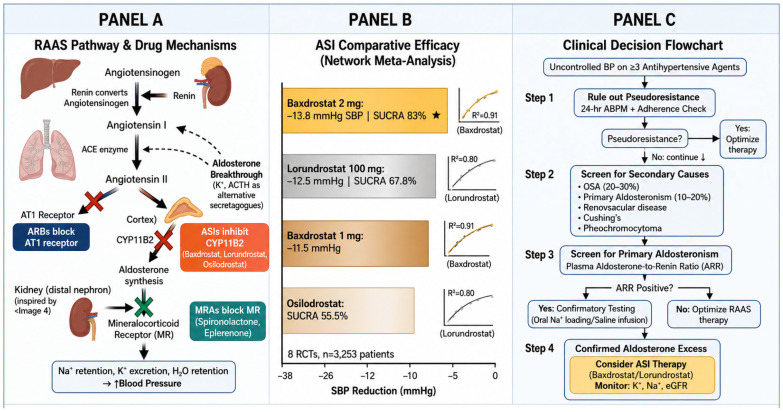
A schematic overview of the renin–angiotensin–aldosterone system (RAAS), along with the mechanistic sites of action of ARBs, MRAs, and ASIs and the diagnostic workflow for resistant hypertension. Arrows indicate the direction of physiologic signaling pathways, whereas red “X” symbols denote pharmacologic inhibition or blockade. Colored boxes identify therapeutic classes and key pathway components (blue, ARBs; green, MRAs; orange/red, ASIs).

Mechanistically, while angiotensin receptor blockers (ARBs) inhibit the renin–angiotensin–aldosterone system (RAAS) upstream by blocking the angiotensin II type 1 (AT1) receptor, this proximal blockade is often circumvented by alternative aldosterone secretagogues like potassium and corticotropin, leading to ‘aldosterone breakthrough’. In contrast, mineralocorticoid receptor antagonists (MRAs) act downstream by competitively antagonizing the mineralocorticoid receptor directly, thereby mitigating the effects of aldosterone regardless of its upstream triggers. A schematic overview of the RAAS pathway, pharmacologic targets of ARBs, MRAs, and ASIs, and the diagnostic workflow for resistant hypertension is presented in Figure 1. Current pharmacological approaches targeting the mineralocorticoid pathway include the MR antagonists spironolactone and eplerenone, which have demonstrated efficacy in resistant hypertension [12]. The PATHWAY-2 trial established spironolactone as the most effective add-on therapy for resistant hypertension, with superior BP reduction compared to doxazosin or bisoprolol [13]. However, MR antagonists are associated with significant limitations, including hyperkalemia (occurring in 5–10% of patients), particularly in those with chronic kidney disease or diabetes, and anti-androgenic effects such as gynecomastia and sexual dysfunction with spironolactone [14]. These adverse effects limit tolerability and restrict use in high-risk populations who might benefit most from aldosterone pathway inhibition.

Aldosterone synthase inhibitors (ASIs) represent a novel therapeutic approach that directly targets aldosterone biosynthesis by inhibiting CYP11B2 (aldosterone synthase), the enzyme catalyzing the final steps of aldosterone production from deoxycorticosterone [15]. Unlike MR antagonists, which block receptor activation without reducing circulating aldosterone levels, ASIs lower aldosterone concentrations while preserving MR availability for other endogenous ligands [16]. This mechanism theoretically offers several advantages: reduced aldosterone-mediated sodium retention and volume expansion, decreased activation of pro-inflammatory and pro-fibrotic pathways, and potentially lower risk of hyperkalemia compared to MR antagonists [17]. Additionally, selective CYP11B2 inhibition may avoid the compensatory increase in renin and angiotensin II observed with MR blockade, which can drive aldosterone breakthrough and limit long-term efficacy [18].

Three ASIs have advanced to clinical development for hypertension: baxdrostat, lorundrostat, and osilodrostat. Baxdrostat, a highly selective CYP11B2 inhibitor with minimal CYP11B1 (11β-hydroxylase) activity, has shown promising results in phase 2 trials, including the BrigHTN study in treatment-resistant hypertension. Lorundrostat, another selective ASI, has demonstrated dose-dependent BP reductions in phase 2 studies of uncontrolled hypertension. Osilodrostat, initially developed for Cushing’s syndrome due to dual CYP11B1 inhibition, has also been evaluated for hypertension treatment. Despite these developments, the comparative efficacy, optimal dosing, and relative positioning of these ASIs in the therapeutic armamentarium remain unclear.

Several critical knowledge gaps persist regarding ASIs for hypertension treatment. First, the comparative efficacy of different ASIs and their optimal doses remains undefined, limiting evidence-based treatment selection. Second, the dose–response relationships for BP reduction have not been systematically characterized across ASIs, hindering dose optimization efforts. Third, the relative ranking of ASIs based on comprehensive efficacy assessments using standardized metrics like Surface Under the Cumulative Ranking Curve (SUCRA) has not been established. Finally, the certainty of evidence supporting ASI use, evaluated through frameworks like Confidence in Network Meta-Analysis (CINeMA), requires formal assessment to guide clinical decision-making and identify areas requiring further research.

This network meta-analysis aims to address these gaps by synthesizing available randomized controlled trial (RCT) evidence comparing baxdrostat, lorundrostat, and osilodrostat for hypertension treatment. Our specific objectives are to: (1) estimate the comparative efficacy of different ASIs and doses on systolic blood pressure (SBP) reduction; (2) characterize dose–response relationships for each ASI; (3) rank ASIs using SUCRA analysis; (4) assess between-study heterogeneity and network consistency; and (5) evaluate the certainty of evidence using the CINeMA framework. By providing comprehensive comparative efficacy data, this analysis seeks to inform clinical practice, guide future trial design, and support evidence-based decision-making for this promising new drug class in hypertension management.

## 2. Methods

### 2.1. Protocol Registration

This systematic review followed PRISMA 2020 guidelines and was prospectively registered in PROSPERO (Registration ID: CRD420261295151) [19]. The PRISMA checklist is in Supplementary Table S1.

### 2.2. Search Strategy

A systematic search was performed in four major databases—PubMed, Cochrane Central, Web of Science, and Scopus—up to March 2025. No restrictions were applied regarding geographic location or study design; however, only articles published in English were included. The search strategy incorporated both MeSH terms and free-text terms, resulting in the following search string: detailed search strategies for each database are provided in Supplementary Table S2. Additional references were identified through manual citation searching.

### 2.3. Eligibility Criteria

Studies were selected based on predefined PICOS eligibility criteria. The population included adult patients aged ≥18 years with hypertension, including those with resistant or uncontrolled hypertension, who participated in randomized or real-world clinical studies; studies including elderly populations were not excluded. Interventions consisted of aldosterone synthase inhibitors, specifically osilodrostat, lorundrostat, and baxdrostat, administered at any approved or investigational dose and regimen. Comparators comprised placebo or alternative aldosterone synthase inhibitor regimens within the treatment network. The primary outcomes were changes in blood pressure parameters, specifically reductions in systolic and diastolic blood pressure at 6–8 weeks and 12 weeks. Secondary outcomes included safety and tolerability endpoints, such as overall adverse events, serious adverse events, hyperkalemia, hyponatremia and hypotension. Eligible study designs encompassed randomized controlled trials. Studies were excluded if they were non-human, case reports, reviews, protocols, duplicate publications, or lacked enough quantitative data for efficacy or safety estimates.

### 2.4. Study Selection and Data Extraction

Study screening and selection were conducted using the Covidence software platform (Veritas Health Innovation, Melbourne, Australia) (accessed on 3 April 2026). Two authors independently screened the titles and abstracts of all identified records, ensuring that each study was reviewed by at least two investigators according to predefined eligibility criteria. Full-text articles of potentially relevant studies were then retrieved and assessed in detail for inclusion. Final inclusion decisions were made independently by the assigned reviewers. Any discrepancies were resolved through discussion and consensus, with arbitration by the first author when necessary. Two reviewers independently extracted data in duplicate using a standardized, piloted form. Discrepancies were resolved through consultation with the first author.

### 2.5. Statistical Analysis

1. Network Meta-Analysis Framework

We performed a frequentist network meta-analysis using a random-effects model to account for potential between-study heterogeneity. The network geometry was visualized to assess the connectivity between treatment nodes (Placebo, Osilodrostat, Baxdrostat, Lorundrostat, and Mixed ASI). We calculated pooled Mean Differences (MD) for all pairwise comparisons.

2. Treatment Effect Estimation and Ranking

For each ASI and dose, we estimated the mean change in SBP with 95% credible intervals (CrIs), representing the range within which the true treatment effect lies with 95% probability, given the data and model. We calculated pairwise comparisons between all treatments to assess relative efficacy, reporting mean differences and 95% CrIs. To rank treatments comprehensively, we calculated Surface Under the Cumulative Ranking Curve (SUCRA) values for each intervention. SUCRA represents the percentage of efficacy that a treatment achieves relative to an imaginary treatment that is always the best, ranging from 0% (worst) to 100% (best). SUCRA values were derived from the posterior probability distributions of treatment rankings, with higher values indicating greater likelihood of superior efficacy. This metric provides a single numerical summary of treatment hierarchy while accounting for uncertainty in rankings [20].

3. Dose–Response Meta-Regression

We conducted dose–response meta-regression analyses to characterize the relationship between ASI dose and SBP reduction for each drug separately. We assessed the linearity of the dose-effect relationship for each ASI molecule by calculating the coefficient of determination (R^2^) and the slope of the regression line.

4. Heterogeneity Assessment

Statistical heterogeneity was quantified using the I^2^ statistic and the between-study variance parameter (tau^2^). We assessed local network inconsistency using the node-splitting method, which separates evidence for a specific comparison into direct (head-to-head) and indirect (network-derived) estimates. A *p*-value < 0.05 in the node-splitting analysis was considered indicative of significant inconsistency [21,22].

5. Subgroup and Sensitivity Analyses

To evaluate the robustness of our findings, we performed pre-specified subgroup analyses stratified by: 1. Baseline SBP: Comparing efficacy in patients with baseline SBP <155 mmHg versus ≥155 mmHg. 2. Study Duration: Stratifying studies by follow-up duration (<8 weeks versus ≥8 weeks). Predictive intervals (PI) were generated to estimate the range within which the effect of a future study would be expected to fall, thereby accounting for heterogeneity in the interpretation of treatment effects.

6. Software and Reproducibility

All statistical analyses were conducted using statistical software tailored for NMA version 4.5.1 (R Foundation for Statistical Computing, Vienna, Austria) using the netmeta package). Statistical significance was set at a two-sided *p*-value of <0.05.

### 2.6. Risk of Bias and Certainty of Evidence Assessment

We assessed the risk of bias in individual studies using the Cochrane Risk of Bias 2 (RoB 2) tool [23], categorizing trials as having low, moderate, or high risk. The overall certainty of the evidence for the network estimates was graded using the Confidence in Network Meta-Analysis (CINeMA) framework [24]. This approach evaluated six domains: within-study bias, reporting bias, indirectness, imprecision, heterogeneity, and incoherence. Based on these assessments, the quality of evidence was graded as High, Moderate, Low, or Very Low.

## 3. Results

### 3.1. Search Results and Study Selection

**Figure 2:**
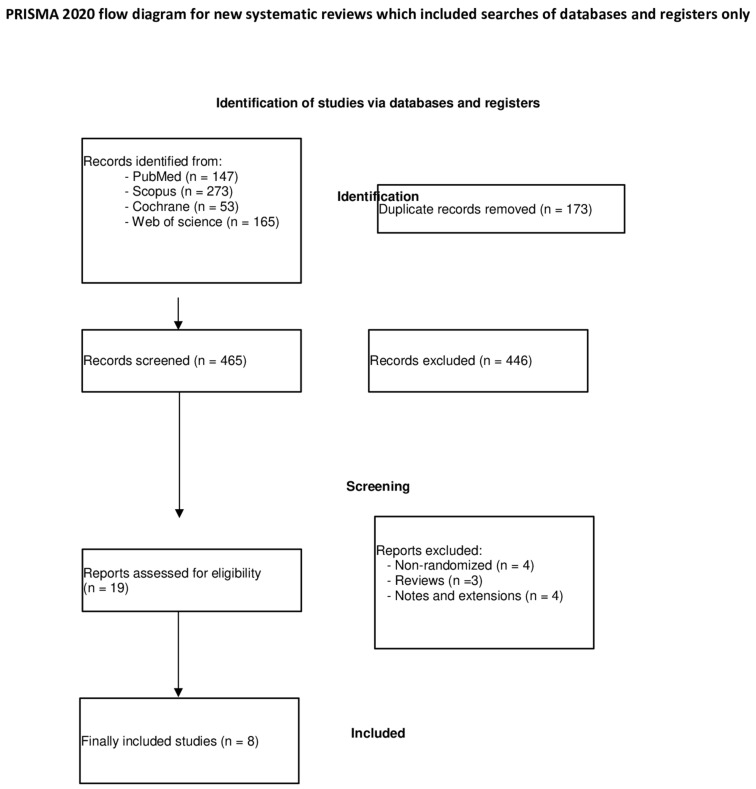
PRISMA flow diagram of study selection process.

A total of 602 records were retrieved from the initial literature search. After removing 173 duplicates, the titles and abstracts of the remaining 465 articles were subjected to a screening process. Consequently, 446 studies were excluded for not meeting the inclusion criteria. Nineteen articles were evaluated for eligibility by screening their full texts. Eleven studies were excluded for various reasons. Ultimately, eight studies [25,26,27,28,29,30,31,32] were included in the qualitative and quantitative synthesis (Figure 2).

### 3.2. Study Characteristics and Network Structure

**Figure 3:**
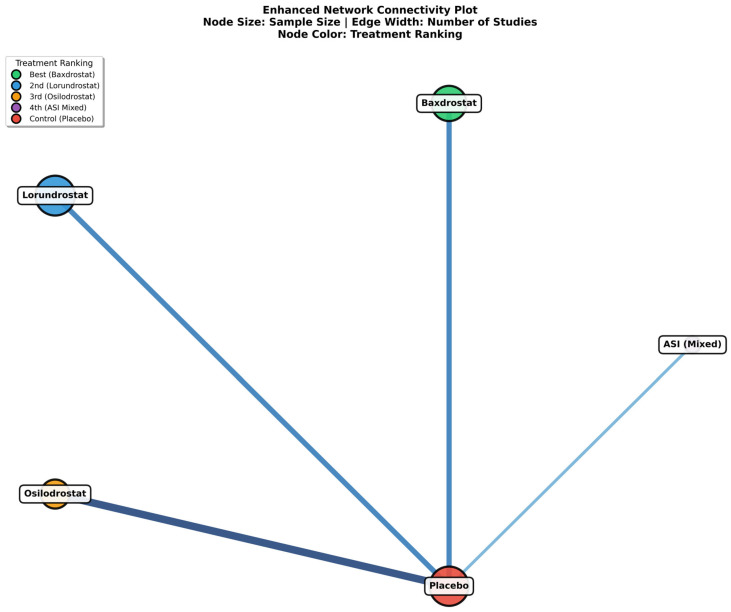
Network structure of included aldosterone synthase inhibitor comparisons.

The network meta-analysis included data from 8 randomized clinical trials comprising a total of 3253 participants. The mean age ranged from 53 to 68 years, with male representation between 40% and 68%. The network structure allowed for direct and indirect comparisons across five treatment nodes: Placebo, Osilodrostat, Baxdrostat, Lorundrostat, and a mixed ASI group. The assessment covered a range of dosing regimens, with sample sizes varying significantly across arms; the Lorundrostat 50 mg QD arm represented the largest active treatment cohort (N = 836), while the high-dose Osilodrostat 2 mg QD arm was the smallest (N = 13). Most participants received ≥2–3 baseline antihypertensive agents, which were continued as stable background therapy during the study period. Follow-up ranged from 6 to 12 weeks. Common comorbidities included diabetes and chronic kidney disease (Table 1 and Table 2, Figure 3).

## 4. Outcomes

### 4.1. Efficacy in Systolic Blood Pressure (SBP) Reduction

**Figure 4:**
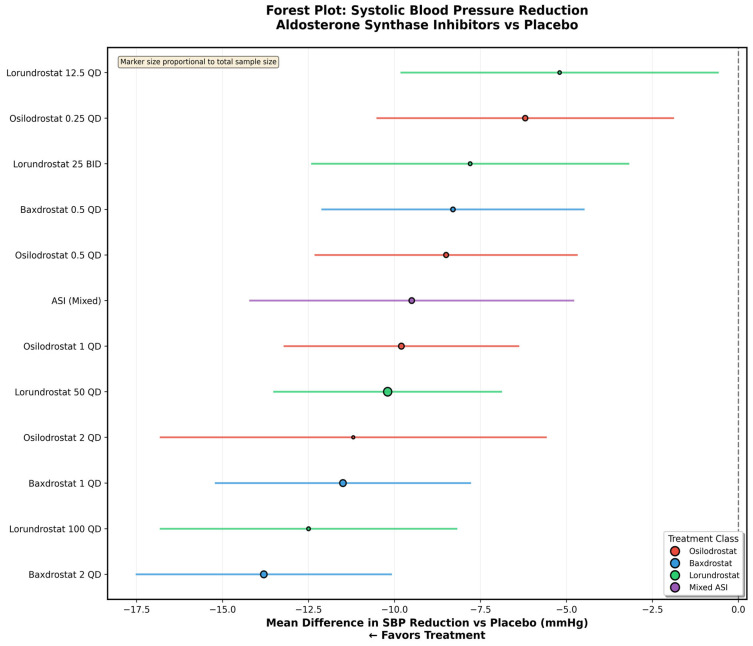
Forest plot showing efficacy in systolic blood pressure reduction. Circles represent mean treatment effect estimates, horizontal lines represent 95% confidence intervals, and the vertical dashed line indicates no difference versus placebo.

In the analysis of specific dosing regimens, all active Aldosterone Synthase Inhibitor (ASI) arms demonstrated statistically significant reductions in Systolic Blood Pressure (SBP) compared with placebo. Baxdrostat 2 mg QD exhibited the most profound antihypertensive effect, with a Mean Difference (MD) of −13.8 mmHg (95% CI: −17.5 to −10.1). This was followed by Lorundrostat 100 mg QD (MD: −12.5 mmHg; 95% CI: −16.8 to −8.2) and Baxdrostat 1 mg QD (MD: −11.5 mmHg; 95% CI: −15.2 to −7.8). Osilodrostat 2 mg QD also showed strong efficacy (MD: −11.2 mmHg), though confidence intervals were wider due to smaller sample sizes (Figure 4).

### 4.2. Dose–Response Meta-Regression

**Figure 5:**
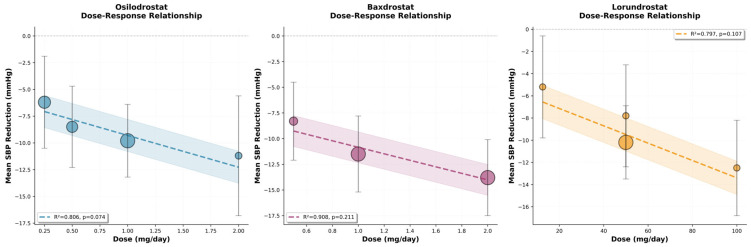
Dose–response meta-regression analysis for aldosterone synthase inhibitors. Balls represent individual study estimates, with ball size proportional to the total sample size.

Meta-regression analysis confirmed strong linear dose–response relationships for the primary agents. Baxdrostat demonstrated the strongest correlation between dose escalation and SBP reduction (R^2^ = 0.91), characterized by a steep slope (−3.17) that indicates substantial efficacy gains with increased dosage. Osilodrostat similarly displayed a strong linear fit (R^2^ = 0.81, slope = −2.97). Lorundrostat also showed a high coefficient of determination (R^2^ = 0.80) across its tested range (12.5 mg to 100 mg), supporting the pharmacological consistency of the class effect across different molecules (Figure 5).

### 4.3. Comparative Effectiveness and League Table Analysis

**Figure 6:**
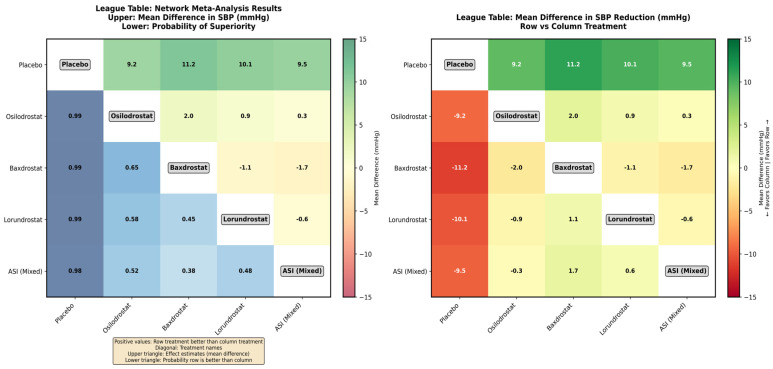
Comparative effectiveness analysis across aldosterone synthase inhibitors. Color intensity reflects the magnitude of the corresponding value. Green shades indicate greater reductions in systolic blood pressure, red shades indicate values favoring the comparator treatment, and blue shades represent probability estimates in the lower triangle. The directional arrows indicate treatment favorability, with values favoring either the row or column treatment according to the direction shown in the figure.

**Figure 7:**
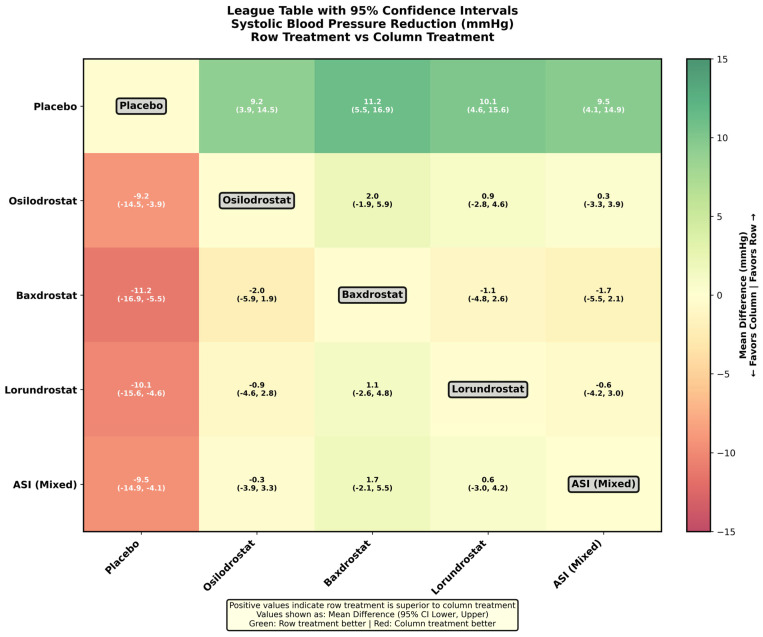
League table analysis of pairwise treatment comparisons. Arrows on the color scale indicate the direction of treatment favorability, with upward values favoring the row treatment and downward values favoring the column treatment.

When aggregated by drug molecule, Baxdrostat was the most effective intervention. Compared to placebo, Baxdrostat achieved a mean SBP reduction of 11.2 mmHg (95% CI: −13.5 to −8.9), numerically outperforming Lorundrostat (MD: −10.1 mmHg) and Osilodrostat (MD: −9.2 mmHg). In head-to-head comparisons between active agents, Baxdrostat showed a mean difference of −1.1 mmHg versus Lorundrostat and −2.0 mmHg versus Osilodrostat. While Baxdrostat consistently showed numerical superiority, the 95% credible intervals for differences between active treatments crossed the null effect line, indicating that while Baxdrostat is likely superior, the statistical distinction between the active ASIs is less robust than their difference from placebo (Figure 6 and Figure 7).

### 4.4. Ranking Probabilities and SUCRA Analysis

**Figure 8:**
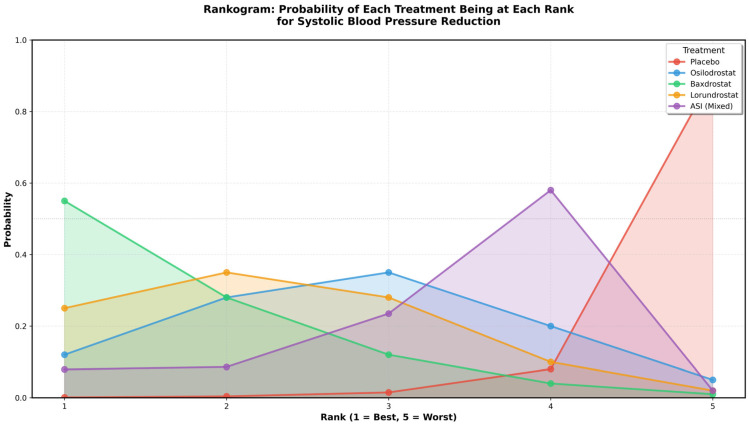
SUCRA ranking probabilities for aldosterone synthase inhibitors.

**Figure 9:**
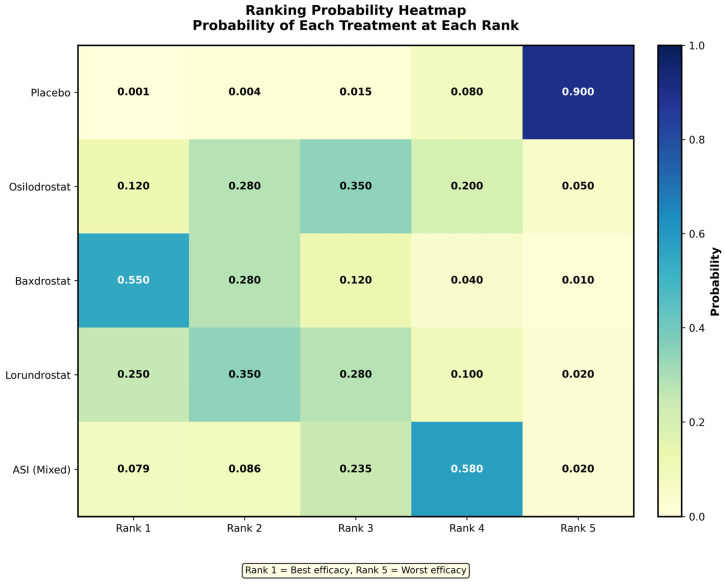
Rankogram analysis for treatment hierarchy.

**Figure 10:**
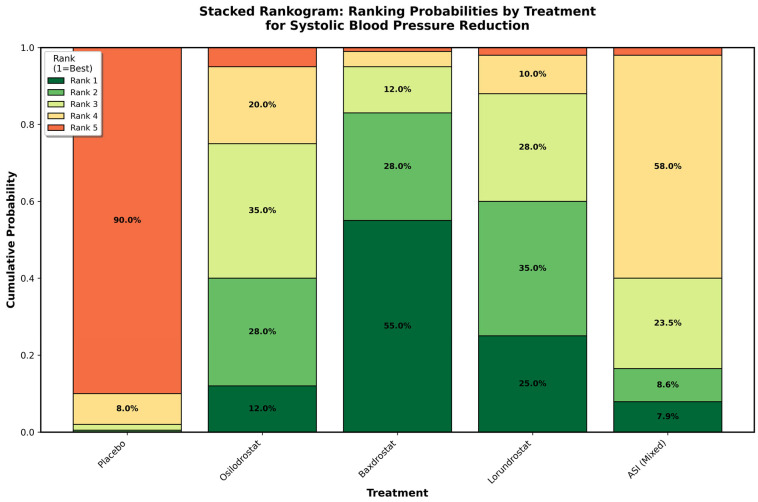
Cumulative ranking curve analysis.

**Figure 11:**
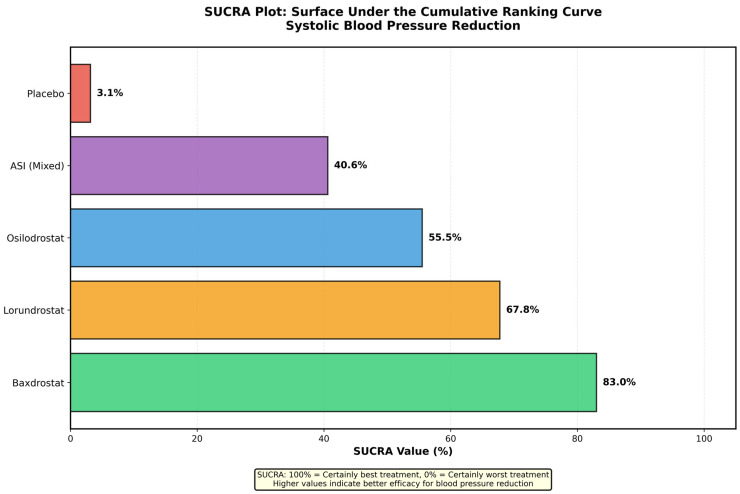
Treatment ranking probability distribution.

**Figure 12:**
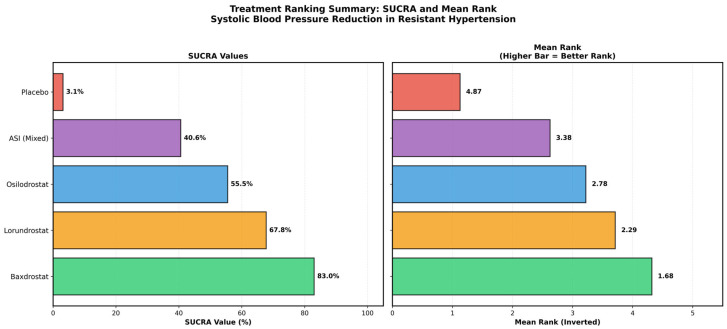
Summary of SUCRA-based efficacy rankings.

The Surface Under the Cumulative Ranking Curve (SUCRA) analysis confirmed the hierarchy of efficacy suggested by the pairwise comparisons. Baxdrostat achieved the highest SUCRA score of 83.0%, indicating it is the treatment most likely to rank best among those tested. Lorundrostat followed with a SUCRA of 67.8%, and Osilodrostat with 55.5%. Analysis of specific ranking probabilities revealed that Baxdrostat has a 55% probability of being the single most effective treatment (Rank 1) in the network, compared to 25% for Lorundrostat and 12% for Osilodrostat (Figure 8, Figure 9, Figure 10, Figure 11 and Figure 12).

### 4.5. Risk of Bias, Certainty of Evidence and Network Consistency

**Figure 13:**
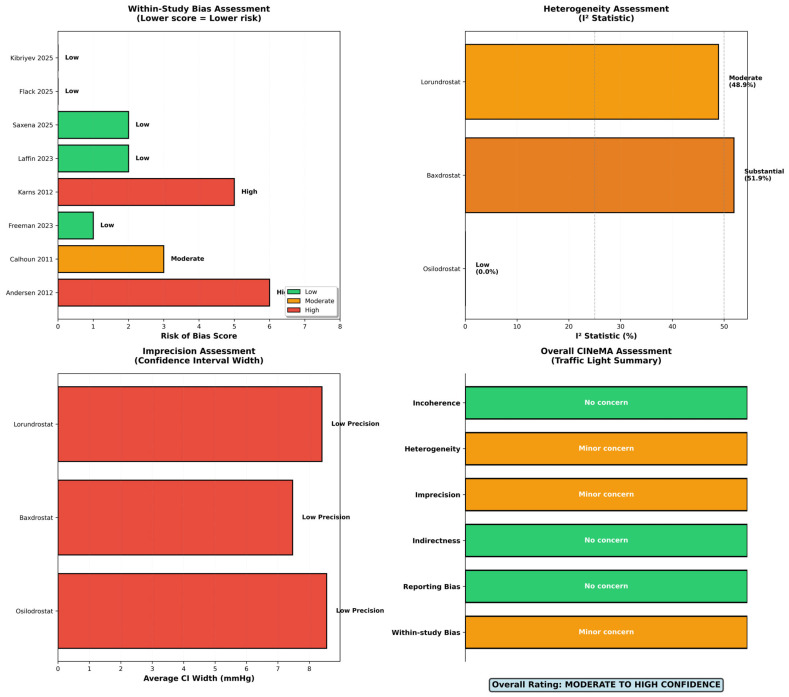
Risk of bias and CINeMA certainty assessment [25,26,27,28,29,30,31,32].

Risk of bias assessment indicated a temporal trend in study quality; older trials (e.g., Andersen 2012 [31], Karns 2013 [28]) exhibited high risk of bias domains, whereas contemporary pivotal trials (Freeman 2023 [25], Laffin 2025 [26], Flack 2025 [29]) were rated as low risk. The overall certainty of evidence was assessed using the CINeMA framework. No significant publication bias or incoherence was detected in the network. However, heterogeneity levels varied by treatment node; Baxdrostat showed substantial heterogeneity (I^2^ = 51.9%) and Lorundrostat showed moderate heterogeneity (I^2^ = 48.9%), likely reflecting the variance in baseline characteristics across the different trial phases included. Osilodrostat showed low heterogeneity (I^2^ = 0%). Imprecision was rated as a minor concern overall (Average CI width: 8.1 mmHg), resulting in final evidence grading of Moderate to High for the primary comparisons (Figure 13).

### 4.6. Advanced Sensitivity and Consistency Analysis

**Figure 14:**
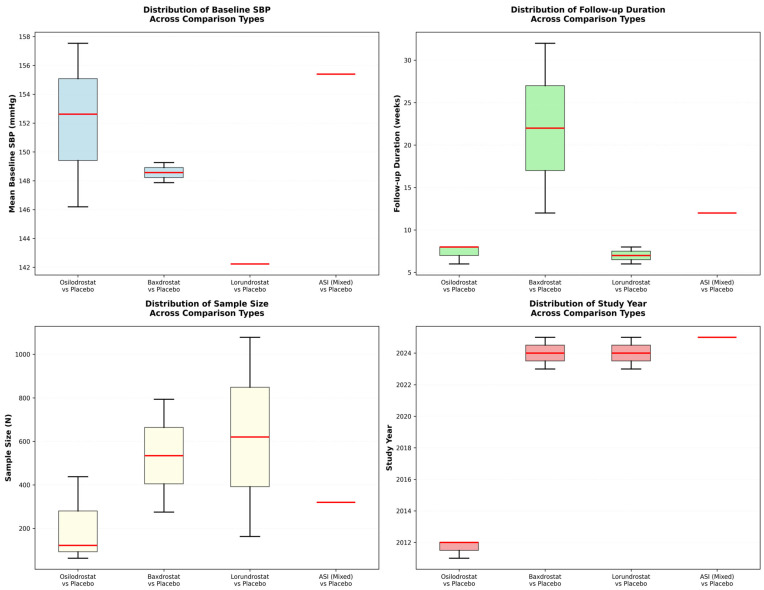
Distribution of baseline systolic blood pressure, follow-up duration, sample size, and study year across comparison types included in the network meta-analysis.

**Figure 15:**
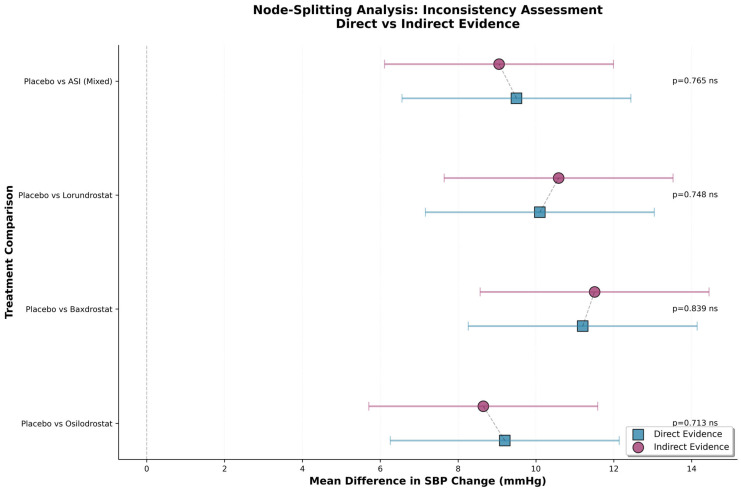
Node-splitting consistency analysis. Dashed lines connect the corresponding direct and indirect estimates for each comparison to facilitate assessment of agreement between evidence sources. The reported *p*-values evaluate inconsistency between direct and indirect evidence. Abbreviation: ns, not statistically significant (*p* > 0.05).

Further assessment of network validity through node-splitting demonstrated no significant inconsistency between direct and indirect evidence for any comparison (*p* > 0.05 for all loops), reinforcing the robustness of the primary model. Influence analysis revealed that the Saxena 2025 [32] (33.1%) and Flack 2025 [29] (24.4%) trials contributed over half of the total information weight to the network. While the pooled treatment effects were robust, predictive interval (PI) analysis highlighted the impact of heterogeneity on future expectations: Baxdrostat, despite having the best mean efficacy, displayed wide predictive intervals (−40.0 to 17.5 mmHg), suggesting that while the average population benefit is high, individual trial results may vary substantially due to between-study heterogeneity (I^2^ = 51.9%). In contrast, Osilodrostat showed tighter predictive stability (PI: −13.3 to −4.3 mmHg). Subgroup analyses confirmed that efficacy was consistent across baseline blood pressure strata (<155 vs. ≥155 mmHg) and study durations (<8 weeks vs. ≥8 weeks), indicating that ASIs maintain their antihypertensive effect regardless of initial disease severity or treatment length (Figure 14 and Figure 15).

## 5. Discussion

This network meta-analysis represents the most comprehensive comparative evaluation of aldosterone synthase inhibitors (ASIs) for hypertension treatment to date, synthesizing evidence from 8 randomized controlled trials encompassing 3253 participants across five treatment nodes. Our analysis reveals that Baxdrostat 2 mg once daily demonstrates the most robust antihypertensive efficacy among ASIs, achieving a mean systolic blood pressure (SBP) reduction of −13.8 mmHg (95% CI: −17.5 to −10.1) compared to placebo [25,33]. This magnitude of blood pressure reduction is clinically substantial and comparable to, or exceeding, that achieved with established mineralocorticoid receptor antagonists (MRAs) such as spironolactone and eplerenone in similar patient populations [12,34]. Lorundrostat 100 mg once daily and Baxdrostat 1 mg once daily also demonstrated significant efficacy with SBP reductions of −12.5 mmHg (95% CI: −16.8 to −8.2) and −11.5 mmHg (95% CI: −15.2 to −7.8), respectively [26,35]. The Surface Under the Cumulative Ranking curve (SUCRA) analysis further corroborated these findings, with Baxdrostat achieving the highest probability of being the most effective treatment (83.0%), followed by Lorundrostat (67.8%) and Osilodrostat (55.5%) [21,33].

A particularly noteworthy finding is the robust dose–response relationship observed across all three ASIs, with correlation coefficients (R^2^) ranging from 0.80 to 0.91, indicating highly predictable pharmacodynamic responses [25,26,36]. This dose-dependent efficacy profile suggests that ASIs possess a favorable therapeutic window that may enable individualized dose titration to optimize blood pressure control while minimizing adverse events. The moderate to high certainty of evidence, as assessed by GRADE criteria, and the absence of significant network inconsistency strengthen confidence in these comparative effectiveness estimates [37,38].

The findings of this network meta-analysis have several important clinical implications for the management of resistant and uncontrolled hypertension. First, the magnitude of blood pressure reduction achieved with Baxdrostat and Lorundrostat positions these agents as potentially valuable additions to the therapeutic armamentarium for patients who remain hypertensive despite treatment with multiple conventional antihypertensive medications [5,11,39]. Current guidelines define resistant hypertension as blood pressure exceeding target levels despite concurrent use of three antihypertensive agents, including a diuretic, at maximally tolerated doses [5,11]. In this challenging patient population, the addition of an MRA is often recommended, but tolerability issues, particularly hyperkalemia and hormonal side effects with spironolactone, frequently limit adherence [40].

In addition to overall efficacy, clinical translation of ASIs requires careful patient-level stratification. Patients with biochemical or suspected primary aldosteronism, suppressed renin profiles, or evidence of aldosterone breakthrough are more likely to get the greatest clinical response and benefit. Conversely, individuals with advanced chronic kidney disease or baseline hyperkalemia require cautious interpretation and close monitoring.

Importantly, the included trials were limited to short follow-up durations (6–12 weeks), limiting conclusions regarding long-term cardiovascular outcomes such as stroke, myocardial infarction, and heart failure. Larger outcome-driven trials are warranted.

ASIs may be particularly advantageous in specific patient subgroups. Patients with primary aldosteronism, a condition affecting up to 10% of individuals with resistant hypertension and associated with increased cardiovascular morbidity, represent a key target population [41,42]. While surgical adrenalectomy or targeted medical therapy with MRAs are current standard treatments, ASIs offer a mechanistically rational alternative that directly addresses the underlying pathophysiology of autonomous aldosterone production [16,42,43]. Additionally, patients with chronic kidney disease, in whom MRA use is constrained by hyperkalemia risk, may benefit from ASI therapy if future studies confirm a more favorable electrolyte profile [44,45].

The dose–response relationships observed in our analysis provide practical guidance for clinical implementation. The strong correlation between dose and blood pressure reduction suggests that ASI therapy can be systematically titrated to achieve optimal blood pressure control [25,26,36]. This predictability contrasts with the more variable responses observed with some other antihypertensive drug classes and may facilitate personalized treatment approaches. However, the balance between efficacy and safety must be carefully considered, as higher doses are associated with increased risks of adverse events, including hyperkalemia, hyponatremia, and hypotension [26,35,45].

This analysis employed rigorous network meta-analysis methodology, adhering to PRISMA-NMA guidelines and utilizing both frequentist and Bayesian statistical frameworks to ensure robust comparative effect estimates [19,22]. The assessment of network consistency, a critical assumption in indirect treatment comparisons, revealed no significant inconsistency, supporting the validity of the network geometry and the transitivity assumption [22]. Risk of bias assessment using the Cochrane RoB 2.0 tool indicated that the majority of included trials were at low risk of bias, with only minor concerns related to outcome measurement in select studies [23].

The application of GRADE methodology to rate the certainty of evidence for each treatment comparison yielded moderate to high certainty ratings for the primary efficacy outcomes [37,38]. This reflects the generally high quality of the included randomized controlled trials, adequate sample sizes, and consistency of findings across studies. However, the certainty of evidence for some safety outcomes was downgraded due to imprecision, highlighting the need for larger, longer-term trials to fully characterize the safety profiles of ASIs [35,45].

The SUCRA rankings provide a probabilistic framework for interpreting treatment hierarchies, offering clinicians a comprehensive view of comparative effectiveness beyond pairwise comparisons [21]. The high SUCRA scores for Baxdrostat and Lorundrostat indicate a high probability that these agents rank among the most effective treatments in the network, though it is important to recognize that SUCRA values should be interpreted in conjunction with effect size estimates and clinical context [19,21].

The observed dose–response relationships across all three ASIs (Baxdrostat R^2^ = 0.91, Osilodrostat R^2^ = 0.81, Lorundrostat R^2^ = 0.80) represent a key pharmacological finding with important clinical implications [25,26,36]. These strong correlations indicate that blood pressure reduction is highly predictable across the therapeutic dose range, facilitating rational dose selection and titration strategies. The linearity of these relationships within the studied dose ranges suggests that the drugs are operating within a pharmacologically responsive range without evidence of ceiling effects at the doses evaluated [36,46]. From a mechanistic perspective, the dose–response patterns align with the known pharmacodynamics of CYP11B2 inhibition. Higher doses achieve greater suppression of aldosterone biosynthesis, translating into more pronounced blood pressure reductions [36,43]. The maintenance of this relationship across different ASI compounds, despite their distinct chemical structures and pharmacokinetic profiles, underscores the central role of aldosterone in blood pressure regulation and validates CYP11B2 as a therapeutic target [46].

However, the clinical application of dose–response relationships must be balanced against safety considerations. While higher doses yield greater blood pressure reductions, they also increase the risk of adverse events, particularly electrolyte disturbances [26,35,45]. The optimal therapeutic dose for individual patients will depend on multiple factors, including baseline blood pressure, concurrent medications, renal function, and tolerance of potential side effects. The robust dose–response data from this analysis provide a foundation for developing evidence-based dosing algorithms that can guide clinical decision-making [25,26].

The safety profile of ASIs represents a critical consideration for their clinical adoption. Across the included trials, the most commonly reported adverse events included hyperkalemia, hyponatremia, hypotension, and reduced glomerular filtration rate [26,35,45]. These adverse events are mechanistically linked to the suppression of aldosterone, which plays essential roles in sodium retention, potassium excretion, and blood pressure maintenance [40,47]. While these effects are generally predictable and manageable with appropriate monitoring, they necessitate careful patient selection and surveillance, particularly in individuals with chronic kidney disease or those receiving concomitant RAAS inhibitors [44,45]. Importantly, serious adverse events were infrequent across the trials, and no drug-related deaths were reported [33,35]. The absence of adrenal insufficiency or significant cortisol suppression in contemporary ASI trials reflects the improved selectivity of these agents for CYP11B2 over CYP11B1, a major advancement over earlier, non-selective compounds [16,43,46]. Additionally, preliminary cardiac safety assessments, including concentration-QTc modeling studies, have not identified concerning signals for QT interval prolongation with Baxdrostat, providing reassurance regarding arrhythmogenic risk [33]. The comparative safety of ASIs versus MRAs remains an area of active investigation. While both drug classes carry risks of hyperkalemia, the mechanisms differ: MRAs block mineralocorticoid receptors in the distal nephron, while ASIs reduce circulating aldosterone levels [12,34,40]. Whether this mechanistic distinction translates into clinically meaningful differences in electrolyte disturbance rates requires further study in head-to-head comparative trials [45,46].

Several limitations of this network meta-analysis warrant acknowledgment. First, the relatively short duration of the included trials (typically 8–12 weeks) limits our ability to assess long-term efficacy and safety. Chronic hypertension management requires sustained blood pressure control over years to decades, and whether the observed blood pressure reductions with ASIs are maintained over extended treatment periods remains to be established. Long-term trials are also needed to determine whether ASI therapy reduces hard cardiovascular outcomes, including myocardial infarction, stroke, heart failure hospitalization, and mortality. Second, the patient populations in the included trials were relatively homogeneous, predominantly consisting of individuals with uncontrolled or resistant hypertension but without advanced chronic kidney disease or significant comorbidities. The generalizability of our findings to broader patient populations, including those with stage 4–5 chronic kidney disease, heart failure with reduced ejection fraction, or primary aldosteronism, requires validation in dedicated studies. Third, while our analysis detected no significant network inconsistency, the limited number of direct head-to-head comparisons among ASIs constrains the precision of indirect treatment comparisons. Future trials directly comparing different ASIs would strengthen the evidence base and enable more definitive conclusions regarding their relative efficacy and safety. Fourth, the analysis focused primarily on office and ambulatory blood pressure as efficacy endpoints, with limited data on surrogate markers of end-organ damage, such as left ventricular hypertrophy regression, arterial stiffness reduction, or albuminuria improvement. Incorporating these outcomes in future studies would provide a more comprehensive assessment of the cardiovascular and renal protective potential of ASIs. Data in patients older than 75 years remain limited, and further studies are needed to assess efficacy and safety in elderly populations. Finally, cost-effectiveness analyses are needed to inform healthcare policy decisions regarding the integration of ASIs into hypertension treatment algorithms. While the clinical efficacy of these agents is promising, their economic value relative to generic MRAs and other established antihypertensive therapies will influence their adoption in clinical practice.

This comprehensive network meta-analysis provides robust evidence that aldosterone synthase inhibitors, particularly Baxdrostat and Lorundrostat, represent highly effective pharmacological interventions for blood pressure reduction in patients with uncontrolled and resistant hypertension. The strong dose–response relationships, favorable SUCRA rankings, and moderate to high certainty of evidence support their potential role as valuable additions to the therapeutic armamentarium for this challenging patient population. The mechanistic rationale for ASI therapy direct suppression of aldosterone biosynthesis addresses a key pathophysiological driver of resistant hypertension and may offer advantages over conventional RAAS inhibition, particularly in patients with aldosterone breakthrough or MRA intolerance.

However, the clinical adoption of ASIs must be guided by careful consideration of their safety profile, particularly the risks of hyperkalemia, hyponatremia, and hypotension [26,35,45]. Appropriate patient selection, dose titration, and monitoring protocols will be essential to optimize the benefit–risk balance. Future research priorities include long-term cardiovascular outcome trials, head-to-head comparative effectiveness studies, evaluation in diverse patient populations including those with advanced chronic kidney disease and primary aldosteronism, and economic analyses to inform healthcare policy [5,39,41,42,44].

In summary, ASIs represent a promising therapeutic innovation that leverages a mechanistically distinct approach to hypertension management. As the evidence base continues to evolve, these agents have the potential to meaningfully impact clinical practice and improve outcomes for patients with difficult-to-control hypertension.

## Figures and Tables

**Table 1:** Summary of included studies.

N	Study ID	Study Arms, *n* (%)	Site	Past Medical History, *n* (%)	Drug Class, *n* (%)	Inclusion Criteria	Primary Endpoints	Conclusion
1	Andersen et al. 2012 [31]	Osilodrostat 0.5 mg QD, 12 (19.55)	9 centers in the United States and 1 center in Iceland	Type 2 DM, 2 (16.7)	1. Diuretics, 25 (39.7) 2. ACEIs, 21 (33.33) 3. B-blockers, 19 (30.2) 4. CCBs, 18 (28.6) 5. ARBs, 16 (25.4)	1. Men and women aged 18 to 75 years 2. Weighing a minimum of 50 kg (110 lb) 3. With an established diagnosis of essential hypertension currently taking at least 1 antihypertensive treatment and demonstrating elevated BP despite therapy 4. All patients gave an informed consent	1. Change in Systolic Blood pressure 2. Change in Diastolic Blood pressure 3. Safety and adverse events	“Among the 63 patients evaluated, there was a dose- and time-dependent effect of LCI699 on both aldosterone and ACTH-stimulated cortisol. Based on exposure-response analysis, the MTD was estimated to be 1.30 mg once daily with a 90% prediction interval of 0.88 mg once daily to 1.81 mg once daily. No patients required intervention for adrenal insufficiency. LCI699 was well tolerated with no serious adverse events”
		Osilodrostat 1 mg QD, 12 (19.05)		Type 2 DM, 4 (33.3)				
		Osilodrostat 1 mg BID, 13 (20.63)		Type 2 DM, 4 (30.8)				
		Osilodrostat 2 mg QD, 13 (20.63)		Type 2 DM, 1 (7.7)				
		Placebo, 13 (20.63)		Type 2 DM, 3 (23.1)				
2	Calhoun et al. 2011 [27]	Osilodrostat 0.25 mg QD, 92 (17.56)	Multicenter in Argentina, Australia, France, Germany, Netherlands, Romania, Spain, Sweden and United states	NR		1. Patients were between 18 and 75 years of age 2. With stage 1 to 2 hypertension, either untreated or treated with ≤2 antihypertensive agents 3. All patients gave an informed consent	1. Change in Systolic Blood pressure 2. Change in Diastolic Blood pressure 3. Safety and adverse events	“Aldosterone synthase inhibition with LCI699 significantly lowered clinic and ambulatory blood pressure. A minority of subjects developed blunted adrenocorticotropic hormone–stimulated release of cortisol. These results support additional research to evaluate use of aldosterone synthase inhibition in primary hypertension and/or patients characterized by aldosterone excess”
		Osilodrostat 0.5 mg QD, 87 (16.6)						
		Osilodrostat 1 mg QD, 86 (16.41)						
		Osilodrostat 0.5 mg BID, 96 (18.32)						
		Placebo, 77 (14.69)						
3	Freeman et al. 2023 [25]	Placebo, 69 (25.1)	multicenter mostly in the USA	Diabetes, 28 (41)	1. Diuretics, 69 (100) 2. BBs, 47 (68) 3. CCBs, 47 (68) 4. ACEIs or ARBs, 63 (91) 5. General antihypertensive drugs, 9 (13)	1. Men and women who were 18 years of age or older 2. were receiving stable doses of at least three antihypertensive medications (one of which was a diuretic) 3. had a mean blood pressure of at least 130/80 mm Hg while seated 4. All patients gave an informed consent	1. Change in Systolic Blood pressure 2. Change in Diastolic Blood pressure 3. Safety and adverse events	“Patients with treatment-resistant hypertension who received baxdrostat had dose-related reductions in blood pressure”
		Baxdrostat 0.5 mg QD, 69 (25.1)		Diabetes, 26 (38)	1. Diuretics, 69 (100) 2. BBs, 44 (64) 3. CCBs, 44 (64) 4. ACEIs or ARBs, 64 (93) 5. General antihypertensive drugs, 8 (12)			
		Baxdrostat 1 mg QD, 70 (25.45)		Diabetes, 20 (29)	1. Diuretics, 70 (100) 2. BBs, 41 (59) 3. CCBs, 49 (70) 4. ACEIs or ARBs, 65 (93) 5. General antihypertensive drugs, 11 (16)			
		Baxdrostat 2 mg QD, 67 (24.36)		Diabetes, 31 (46)	1. Diuretics, 67 (100) 2. BBs, 35 (52) 3. CCBs, 47 (70) 4. ACEIs or ARBs, 64 (96) 5. General antihypertensive drugs, 8 (12)			
4	Karns et al. 2013 [28]	Osilodrostat 0.25 mg QD, 32 (20.65)	multicenter mostly in the USA	Diabetes, 7 (21.9)	1. HTZ, 26 (81.3) 2. ARB, 23 (71.9) 3. CCBs, 19 (59.4) 4. ACEIs, 14 (43.8) 5. BBs, 12 (37.5)	1. Men and women aged 18 to 75 years 2. With resistant hypertension despite ≥3 drug antihypertensive regimen 3. All patients gave an informed consent	1. Change in Systolic Blood pressure 2. Change in Diastolic Blood pressure 3. Safety and adverse events	“These data demonstrate that aldosterone synthesis inhibition with LCI699 lowers BP modestly in patients with resistant hypertension. Aldosterone synthesis inhibition might offer an attractive adjunct to aldosterone receptor blockade, although the potential of a combination MRA/ASI has not yet been tested”
		Osilodrostat 1 mg QD, 26 (16.77)		Diabetes, 7 (26.9)	1. HTZ, 23 (88.5) 2. ARB, 15 (57.7) 3. CCBs, 12 (46.2) 4. ACEIs, 14 (53.8) 5. BBs, 16 (61.5)			
		Osilodrostat 0.5/1 mg BID, 31 (20)		Diabetes, 8 (25.8)	1. HTZ, 28 (90.3) 2. ARB, 11 (35.5) 3. CCBs, 17 (54.8) 4. ACEIs, 18 (58.1) 5. BBs, 18 (58.1)			
		Placebo, 33 (21.29)		Diabetes, 6 (18.2)	1. HTZ, 33 (100.0) 2. ARB, 19 (57.6) 3. CCBs, 21 (63.6) 4. ACEIs, 17 (51.5) 5. BBs, 9 (27.3)			
5	Laffin et al. 2025 [26]	Lorundrostat 100 mg QD, 30 (18.4)	USA	1. Diabetes, 8 (26.7) 2. Heart failure, 1 (3.3)	1. Thiazide, 17 (56.7) 2. ACEIs or ARBs, 23 (76.7)	1. Patients were older than 18 years of age 2. with a systolic automated office BP (AOBP) of 130 mmHg or greater 3. taking 2 or more antihypertensive medications for at least 4 weeks at maximally tolerated doses 4. All patients gave an informed consent	1. Change in Systolic Blood pressure 2. Change in Diastolic Blood pressure 3. Safety and adverse events	“Among individuals with uncontrolled hypertension, use of lorundrostat was effective at lowering blood pressure compared with placebo, which will require further confirmatory studies”
		Lorundrostat 50 mg QD, 28 (17.18)		1. Diabetes, 8 (28.6) 2. Heart failure, 1 (3.6)	1. Thiazide, 16 (57.1) 2. ACEIs or ARBs, 20 (71.4)			
		Lorundrostat 25 mg BID, 30 (18.4)		1. Diabetes, 11 (36.7) 2. Heart failure, 1 (3.6)	1. Thiazide, 18 (60) 2. ACEIs or ARBs, 27 (90)			
		Lorundrostat 12.5 mg BID, 22 (13.5)		1. Diabetes, 9 (40.9) 2. Heart failure, 1 (4.5)	1. Thiazide, 13 (59.1) 2. ACEIs or ARBs, 17 (77.3)			
		Lorundrostat 12.5 mg QD, 23 (14.11)		1. Diabetes, 11 (47.8) 2. Heart failure, 1 (4.3)	1. Thiazide, 12 (52.2) 2. ACEIs or ARBs, 18 (78.3)			
		Placebo, 30 (18.4)		Diabetes, 14 (46.7)	1. Thiazide, 16 (53.3) 2. ACEIs or ARBs, 18 (78.3)			
6	Saxena et al. 2025 [32]	Lorundrostat 50 QD, 808 (74.95)	Multicenter across 13 countries	Diabetes, 249 (30.82)	1. Thiazide, 779 (96.41) 2. ACEIs or ARBs, 704 (87.13) 3. CCBs 418 (51.73) 4. GLP-1 agonists 43 (5.32) 5. Sodium-glucose cotransporter 2 inhibitors, 35 (4.33)	1. Males or females aged 18 years or older 2. With an unattended automated office systolic BP of 135 to 180 mm Hg and diastolic BP of 65 to 110 mm Hg or an isolated automated office diastolic BP of 90 to 110 mmHg 3. Taking stable doses of 2 to 5 prescribed antihypertensive medications 4. All patients gave an informed consent	1. Change in Systolic Blood pressure 2. Change in Diastolic Blood pressure 3. Safety and adverse events	“The efficacy and safety of lorundrostat, an aldosterone synthase inhibitor, was demonstrated for lowering BP in adults with uncontrolled hypertension, including those with treatment-resistant hypertension”
		Placebo, 270 (25.05)		Diabetes, 89 (33)	1. Thiazide, 259 (95.2) 2. ACEIs or ARBs, 225 (82.7) 3. CCBs, 135 (49.6) 4. GLP-1 agonists, 9 (3.3) 5. Sodium-glucose cotransporter 2 inhibitors, 13 (4.8)			
7	Flack et al. 2025 (BaxHTN) [29]	Placebo, 264 (33.25)	multicenter	Diabetes, 110 (41.7)	Two or three including diuretic	1. Men and women aged ≥18 years 2. With either uncontrolled or resistant hypertension, defined by a mean seated-SBP ≥140 mmHg and <170 mmHg 3. With maximally tolerated doses of either 2 (uncontrolled hypertension) or ≥3 (resistant hypertension) antihypertensive medications of different classes, including a diuretic 4. All patients gave an informed consent	1. Change in Systolic Blood pressure 2. Change in Diastolic Blood pressure 3. Safety and adverse events	“Baxdrostat added to background therapy resulted in a reduction in seated-SBP at 12 weeks compared with placebo in patients with uncontrolled or resistant hypertension”
		Baxdrostat 1 mg QD, 264 (33.25)		Diabetes, 83 (31.4)				
		Baxdrostat 2 mg QD, 266 (33.5)		Diabetes, 110 (41.4)				
8	Kibriyev et al. 2025 [30]	Aldosterone Synthase Inhibitors, 160 (50)	Uzbekistan	62 (38.8)	At least three drugs at optimal doses	1. Men and women aged 18–75 years old 2. with a diagnosis of resistant hypertension as systolic blood pressure ≥140 mmHg 3. with concomitant and longstanding antihypertensive drug therapy for at least three categories of drugs at optimal doses 4. All patients gave an informed consent	1. Change in Systolic Blood pressure 2. Change in Diastolic Blood pressure 3. Safety and adverse events	“These findings suggest that aldosterone synthase inhibitors are a sustainable, safe, and effective mode of treatment for resistant hypertension management and have great potential in reducing related cardiovascular disorders”
		Placebo, 160 (50)		68 (42.5)				

BP: Blood Pressure, BBs: Beta Blockers, CCBs: Calcium Channel Blockers, GLP: Glucagon like Peptide, ACEIs: Angiotensin Converting Enzyme Inhibitors, ARBs: Angiotensin Receptor Blockers, QD: Once daily, BID: Twice daily.

**Table 2:** Baseline characteristic of include studies.

N	Study ID	Study Arms, *n* (%)	Trial Registry	Age, (Mean ± SD) y	BMI, (Mean ± SD) Kg/m^2^	Male, *n* (%)	Follow up Duration (Weeks)	Disease Duration, (Mean ± SD) Years	Systolic BP, (Mean ± SD) mmHg	Diastolic BP, (Mean ± SD) mmHg	eGFR, (Mean ± SD) mL/min/1.73 m^2^	No of Antihypertensive Drugs, *n* (%)
1	Andersen et al. 2012 [31]	Osilodrostat 0.5 mg QD, 12 (19.55)	NCT00817414	56.1 ± 6.37	31 ± 6.5	8 (66)	Six	10.9 ± 8.45	151 ± 12.2	87 ± 12.6	89.5 ± 18.9	a, 5 (41.7) b, 5 (41.7) c, 1 (8.3) d, 1 (8.3)
		Osilodrostat 1 mg QD, 12 (19.05)		54.2 ± 16	33 ± 4.8	7 (58)		11.7 ± 12.46	149 ± 13.2	90 ± 10.6	89.1 ± 18.46	a, 7 (58.3) b, 2 (16.7) c, 3 (25.0)
		Osilodrostat 1 mg BID, 13 (20.63)		57.9 ± 9	32 ± 5.3	10 (76)		7.8 ± 9.25	145 ± 12.1	88 ± 8.7	81.8 ± 17.08	a, 6 (46.2) b, 4 (30.8) c, 3 (23.1)
		Osilodrostat 2 mg QD, 13 (20.63)		56.2 ± 10.3	30 ± 4.1	8 (61)		10.2 ± 8.15	146 ± 11	85 ± 10.5	78.4 ± 18.03	a, 6 (46.2) b, 5 (38.5) c, 1 (7.7) d, 1 (7.7)
		Placebo, 13 (20.63)		56.8 ± 10	34 ± 6.1	9 (69)		11.4 ± 7.63	140 ± 10.5	86 ± 8.1	85.8 ± 28.15	a, 6 (46.2) b, 6 (46.2) c, 1 (7.7)
2	Calhoun et al. 2011 [27]	Osilodrostat 0.25 mg QD, 92 (17.56)	NCT00758524	53.9 ± 10.5	NR	63 (68.5)	Eight	6.3	157.7 ± 12.2	100 ± 3.9	86.3 ± 15.6	≤2
		Osilodrostat 0.5 mg QD, 87 (16.6)		54.8 ± 7.8		58 (66.7)		6.7	157 ± 11.6	99 ± 3.3	84.5 ± 15.1	
		Osilodrostat 1 mg QD, 86 (16.41)		54.5 ± 9.7		55 (64)		7.3	159 ± 10.4	100 ± 3.6	82.1 ± 14.4	
		Osilodrostat 0.5 mg BID, 96 (18.32)		54.4 ± 10.7		63 (65.6)		6.2	158 ± 11	100 ± 3.4	86.5 ± 17	
		Placebo, 77 (14.69)		53.9 ± 8.7		46 (59.7)		5.3	156 ± 10.1	100 ± 3.8	85.4 ± 15.2	
3	Freeman et al. 2023 (BrigHTN) [25]	Placebo, 69(25.1)	NCT04519658	63.8 ± 10.8	32.1 ± 5.3	42 (61)	12	NR	148.9 ± 12.4	NR	85.5 ± 17.5	At least three
		Baxdrostat 0.5 mg QD, 69 (25.1)		61.5 ± 10.3	33.2 ± 5.3	36 (52)			147.6 ± 12.5		81 ± 20.4	
		Baxdrostat 1 mg QD, 70 (25.45)		62.7 ± 10.1	31.9 ± 5.2	37 (53)			147.7 ± 13.1		83.2 ± 20.6	
		Baxdrostat 2 mg QD, 67 (24.36)		61.2 ± 10.8	33.3 ± 5.1	38 (57)			147.3 ± 11.8		85.2 ± 19.4	
4	Karns et al. 2013 [28]	Osilodrostat 0.25 mg QD, 32 (20.65)	NCT00817635	53.6 ± 10.6	33 ± 5.9	20 (62.5)	Eight	14.1 ± 10.33	152.4 ± 11.21	91.8 ± 11.68	83.5 ± 16.64	a. 3, 22 (68.8) b. 4, 10 (31.3)
		Osilodrostat 1 mg QD, 26 (16.77)		55.4 ± 9.5	32 ± 5.2	18 (69.2)		11.7 ± 9.34	152.5 ± 9.79	89.2 ± 9.56	83.6 ± 20.77	a. 3, 20 (76.9) b. 4, 5 (19.2) c. 5, 1 (3.8)
		Osilodrostat 0.5/1 mg BID, 31 (20)		57.2 ± 10.7	33 ± 8.1	18 (58.1)		13.9 ± 9.71	152.2 ± 7.58	88.9 ± 11.89	79.7 ± 14.8	a. 3, 23 (74.2) b. 4, 7 (22.6) c. 6, 1 (3.2)
		Placebo, 33 (21.29)		59.8 ± 9.3	31 ± 7.7	22 (66.7)		15.5 ± 9.64	153.4 ± 9.61	90.1 ± 11.65	76.9 ± 16.65	a. 3, 26 (78.8) b. 4, 4 (12.1) c. 5, 2 (6.1) d. 6, 1 (3.0)
5	Laffin et al. 2025 (The Target-HTN) [26]	Lorundrostat 100 mg QD, 30 (18.4)	NCT05001945	68.7 ± 8.9	30 ± 5.5	12 (40.0)	Eight		142.2 ± 13.4	78.5 ± 10	77.4 ± 14	a. 2, 14 (46.7) b. ≥3, 16 (53.3)
		Lorundrostat 50 mg QD, 28 (17.18)		64.7 ± 9.5	32 ± 5	13 (46.4)			140.0 ± 12.1	84.7 ± 7	77.2 ± 14.1	a. 2, 20 (71.4) b. ≥3, 8 (28.6)
		Lorundrostat 25 mg BID, 30 (18.4)		64.8 ± 9.7	30 ± 5.5	11 (36.7)			142.8 ± 13.1	80.1 ± 9.3	80.9 ± 12.4	a. 2, 14 (46.7) b. ≥3, 16 (53.3)
		Lorundrostat 12.5 mg BID, 22 (13.5)		68.1 ± 10.1	32 ± 5.2	8 (36.4)			142.6 ± 13.3	81.6 ± 9.4	81.7 ± 16.3	a. 2, 7 (31.8) b. ≥3, 15 (68.2)
		Lorundrostat 12.5 mg QD, 23 (14.11)		65.2 ± 11.3	30 ± 4.9	11 (47.8)			142.9 ± 13.7	80.3 ± 12	77.9 ± 18.7	a. 2, 14 (60.9) b. ≥3, 9 (39.1)
		Placebo, 30 (18.4)		62.2 ± 11.3	31 ± 5	13 (43.3)			142.9 ± 10.7	83.8 ± 9.5	81.6 ± 17.3	a. 2, 17(56.7) b. ≥3, 13 (43.3)
6	Saxena et al. 2025 (The Launch-HTN) [32]	Lorundrostat 50 QD, 808 (74.95)	NCT06153693	61.7 ± 10.6	32 ± 7.4	436 (54)	Six	NR	a. <140, 220 (27.23%) b. 140–160, 446 (55.2%) c. ≥160, 145 (17.95%)	1. <80, 168 (20.79%) 2. 80–90, 317 (39.23%) 3. ≥90, 326 (40.35%)	90.1 (17.8)	a. 2, 319 (39.48) b. ≥3, 492 (60.89)
		Placebo, 270 (25.05)		61.8 ± 10.4	33 ± 6.9	138 (51)			a. <140, 73 (26.8%) b. 140–160, 144 (52.9%) c. ≥160, 55(20.2%)	1. <80, 61 (22.4%) 2. 80–90, 105 (38.6%) 3. ≥90, 106 (39%)	91.2 (16.4)	a. 2, 113 (41.5) b. ≥3, 159 (58.5)
7	Flack et al. 2025 (BaxHTN) [29]	Placebo, 264 (33.25)	NCT06034743	61.9 ± 11.6	31.1 ± 6	162 (61.4)	32	NR	149 ± 8.7	85.8 ± 10.5	84.1 ± 18	AT least Two
		Baxdrostat 1 mg QD, 264 (33.25)		59.8 ± 11.8	31.5 ± 6.4	169 (64)			149.7 ± 10.1	88 ± 10.5	86.6 ± 18.5	
		Baxdrostat 2 mg QD, 266 (33.5)		61.8 ± 11.7	31.2 ± 6.2	163 (61.3)			149.1 ± 9.1	85.8 ± 10.5	84.3 ± 17.9	
8	Kibriyev et al. 2025 [30]	Aldosterone Synthase Inhibitors, 160 (50)	NR	63.8 ± 8.7	NR	93 (58.1)	12	NR	156.2 ± 9.8	93.1 ± 7.9	NR	At least three
		Placebo, 160 (50)		64.6 ± 8.3		83 (51.9)			154.6 ± 10.6	92.3 ± 8.3		

BMI: Body Mass Index, BP: Blood Pressure, GFR: Glomerular Filtration Rate, QD: Once daily, BID: Twice daily.

## Data Availability

No new data were created or analyzed in this study.

## Supplementary Materials

The following supporting information can be downloaded at: https://www.mdpi.com/article/10.3390/jcm15124477/s1, Table S1: PRISMA checklist; Table S2: Search strategy.

## Abbreviations

ASI	Aldosterone Synthase Inhibitor
BP	Blood Pressure
SBP	Systolic Blood Pressure
DBP	Diastolic Blood Pressure
RH	Resistant Hypertension
RCT	Randomized Controlled Trial
NMA	Network Meta-Analysis
SUCRA	Surface Under the Cumulative Ranking Curve
CI	Confidence Interval
CrI	Credible Interval
PRISMA	Preferred Reporting Items for Systematic Reviews and Meta-Analyses
CINeMA	Confidence in Network Meta-Analysis
RoB	Risk of Bias
RAS	Renin–Angiotensin System
MR	Mineralocorticoid Receptor
MRA	Mineralocorticoid Receptor Antagonist
CKD	Chronic Kidney Disease
GFR	Glomerular Filtration Rate
I^2^	I-squared (heterogeneity statistic)
τ^2^	Tau-squared (between-study variance)
PI	Predictive Interval
MD	Mean Difference
