# Supplementary material for: Dose–Response Relationships and Comparative Efficacy of Aldosterone Synthase Inhibitors in Resistant Hypertension: A Comprehensive Network Meta-Analysis and Meta-Regression
Source: J Clin Med. 2026 Jun 9;15(12):4477. doi: 10.3390/jcm15124477 (PMC13301991; doi:10.3390/jcm15124477)
Supplement: Supplementary file 1 [file jcm-15-04477-s001.zip › jcm-4270893-supplementary.pdf]

Table S1: PRISMA Checklist

Section and Topic	Item #	Checklist item	Location where item is reported
**TITLE**			
Title	1	Dose-Response Relationships and Comparative Efficacy of Aldosterone Synthase Inhibitors in Resistant Hypertension: A Comprehensive Network Meta-Analysis and Meta-Regression	Page 1 – Title page
**ABSTRACT**			
Abstract	2	The abstract provides a concise summary of your systematic review, including background, objectives, methods, results, and conclusions.	Page 1-2<br>Abstract
**INTRODUCTION**			
Rationale	3	The rationale is provided in the introduction, highlighting the challenge of resistant hypertension and the novel role of aldosterone synthase inhibitors (ASIs)	Pages 2–3 – Introduction
Objectives	4	The objective is clearly stated: to estimate the comparative efficacy of ASIs and doses on systolic blood pressure reduction	Page 4 – Last paragraph of Introduction
**METHODS**			
Eligibility criteria	5	The inclusion criteria (adults with hypertension, RCTs, specific ASIs) and exclusion criteria (non-human studies, case reports, reviews) are defined in the manuscript.	Pages 5<br>Section 2.3 Eligibility Criteria
Information sources	6	Databases used include PubMed, Web of Science, CENTRAL, and Scopus. The last search date is up to March 2025 .	Page 5 – Section 2.2 Search Strategy
Search strategy	7	The manuscript provides the search strategy, which includes both MeSH and free-text terms .	Page 5 – Section 2.2 and Supplementary Table S1
Selection process	8	Studies were screened by two independent reviewers using the Covidence software, and discrepancies were resolved through discussion .	Page 5 – Section 2.4 Study Selection and Data Extraction
Data collection process	9	Data extraction was conducted independently by two reviewers.	Page 5 – Section 2.4 Study Selection and Data Extraction
Data items	10a	Primary outcome: systolic blood pressure (SBP) reduction.	Pages 5 Primary outcomes<br>Section 2.3 Eligibility Criteria
	10b	Secondary outcomes include safety and adverse events.	Pages 5 Secondary outcomes and safety endpoints<br>Section 2.3 Eligibility Criteria
Study risk of bias assessment	11	The risk of bias was assessed using the RoB 2 tool.	Page 6 – Section 2.6 Risk of Bias and Certainty Assessment
Effect measures	12	The effect measure used is the mean difference (MD) in SBP reduction, with 95% confidence intervals (CIs)	Page 5 – 6

Section and Topic	Item #	Checklist item	Location where item is reported
			Statistical Analysis
Synthesis methods	13a	Studies were grouped by ASI types (Baxdrostat, Lorundrostat, Osilodrostat) and doses for direct and indirect comparisons.	Page 6 – Network Meta-Analysis Framework
	13b	Missing data were handled by using available data and standardizing outcomes, with clarifications sought when necessary.	Page 6
	13c	Results were tabulated in structured tables and displayed using forest plots, league tables, and SUCRA ranking plots.	Pages 6 Tables and Figures
	13d	A frequentist random-effects model was used for meta-analysis, with I^2 statistics to assess heterogeneity. Analyses were conducted using R version 4.5.1 and the netmeta package.	Page 6 – Random-effects model and heterogeneity analysis
	13e	Meta-regression was performed to explore dose-response relationships, and subgroup analyses were conducted based on baseline SBP and study duration.	Page 6 – Meta-regression and subgroup analyses
	13f	Sensitivity analyses included excluding high-risk bias studies and node-splitting to assess network consistency.	Pages 6<br><br>Sensitivity and consistency analyses
Reporting bias assessment	14	The risk of bias due to missing results is indirectly addressed through the RoB 2 tool .	Pages 6 – CINeMA and bias assessment
Certainty assessment	15	Certainty of evidence was assessed using the CINeMA framework	Page 6 – CINeMA framework
**RESULTS**			
Study selection	16a	The search identified 602 records, of which 8 studies were included in the analysis.	Page 6 – Search Results and Figure 1
	16b	Studies that were excluded are listed, with reasons for exclusion provided .	Page 6 – Excluded studies and Figure 1
Study characteristics	17	The characteristics of the 8 included studies, such as study design and sample size, are summarized in Tables 1 and 2 .	Pages 7 – Tables 1 and 2
Risk of bias in studies	18	The risk of bias for each study is presented in the "Risk of Bias in Studies" section .	Page 22 – 4.5.Risk of Bias section
Results of individual studies	19	Summary statistics and effect estimates are presented for SBP reduction across all studies .	Pages 15– 21<br><br>4.1.Outcome analyses
Results of syntheses	20a	This is summarized in the "Study Characteristics" and "Risk of Bias" sections .	Pages 7-14 – Study characteristics
	20b	The results of the network meta-analysis are presented, including effect sizes and 95% CIs for each treatment .	Pages 15-25 – Comparative efficacy analyses
	20c	The heterogeneity results using I^2 statistics are included .	Page 23 – Heterogeneity assessment
	20d	Sensitivity analyses are included to assess the robustness of the results .	Page 23 – Sensitivity analyses
Reporting biases	21	While not explicitly stated, the use of RoB 2 helps assess this risk .	Page 22 – 4.5.Risk of Bias section

Section and Topic	Item #	Checklist item	Location where item is reported
Certainty of evidence	22	The certainty of evidence is presented using the CINeMA framework	Page 22 – 4.5.Risk of Bias section
**DISCUSSION**			
Discussion	23a	The discussion interprets the results in the context of previous studies on ASIs .	Pages 25-26 – Discussion
	23b	Limitations such as short study durations and small sample sizes are discussed .	Pages 27 – Limitations
	23c	Limitations related to the review process, such as the lack of long-term data, are addressed .	Pages 28 – Review limitations
	23d	The implications for clinical practice, policy, and future research are discussed in the "Discussion" section .	Pages 26-27 – Clinical implications and future directions
**OTHER INFORMATION**			
Registration and protocol	24a	The review was registered in PROSPERO (Registration ID: CRD420261295151) .	Page 5 – Protocol Registration
	24b	The protocol can be accessed via the PROSPERO database .	Page 5 – PROSPERO registration
	24c	No amendments to the protocol are mentioned .	Not reported
Support	25	No funding was received for this review .	Page 29 – Funding statement
Competing interests	26	The authors declare no competing interests .	Page 29 – Conflicts of Interest
Availability of data, code and other materials	27	Data supporting the findings are available upon reasonable request .	Page 29 – Data Availability Statement

Table S2: Search strategy.

Database	Search Terms	Search Field	Search Results
Pubmed	(("aldosterone synthase inhibitor" OR "aldosterone synthase inhibitors" OR "aldosterone synthase" OR "CYP11B2 inhibitor" OR "CYP11B2 inhibitors" OR "aldosterone synthase inhibitor*" OR "CYP11B2") AND (hypertens* OR "resistant hypertens*" OR "refractory hypertens*" OR "treatment-resistant hypertens*" OR "drug-resistant hypertens*" OR "high blood pressure") AND (Random* OR "randomized controlled triaL" OR randomized OR randomised OR randomly OR trial OR "clinical trial")) ("aldosterone synthase inhibitor"[All Fields] OR "aldosterone synthase inhibitors"[All Fields] OR "aldosterone synthase"[All Fields] OR "CYP11B2 inhibitor"[All Fields] OR "CYP11B2 inhibitors"[All Fields] OR "aldosterone synthase inhibitor*"[All Fields] OR "CYP11B2"[All Fields]) AND ("hypertens*"[All Fields] OR "resistant hypertens*"[All Fields] OR "refractory hypertens*"[All Fields] OR "treatment resistant hypertens*"[All Fields] OR "drug resistant hypertens*"[All Fields] OR "high blood pressure"[All Fields]) AND ("random*"[All Fields] OR "randomized controlled triaL"[All Fields] OR ("random allocation"[MeSH Terms] OR ("random"[All Fields] AND "allocation"[All Fields]) OR "random allocation"[All Fields] OR "randomization"[All Fields] OR "randomized"[All Fields] OR "random"[All Fields] OR "randomisation"[All Fields] OR "randomisations"[All Fields] OR "randomise"[All Fields] OR "randomised"[All Fields] OR "randomising"[All Fields] OR "randomizations"[All Fields] OR "randomize"[All Fields] OR "randomizes"[All Fields] OR "randomizing"[All Fields] OR "randomness"[All Fields] OR "randoms"[All Fields]) OR ("random allocation"[MeSH Terms] OR ("random"[All Fields] AND "allocation"[All Fields]) OR "random allocation"[All Fields] OR "randomization"[All Fields] OR "randomized"[All Fields] OR "random"[All Fields] OR "randomisation"[All Fields] OR "randomisations"[All Fields] OR "randomise"[All Fields] OR "randomised"[All Fields] OR "randomising"[All Fields] OR "randomizations"[All Fields] OR "randomize"[All Fields] OR "randomizes"[All Fields] OR "randomizing"[All Fields] OR "randomness"[All Fields] OR "randoms"[All Fields]) OR "randomly"[All Fields] OR ("clinical trials as topic"[MeSH Terms] OR ("clinical"[All Fields] AND "trials"[All Fields] AND "topic"[All Fields]) OR "clinical trials as topic"[All Fields] OR "trial"[All Fields] OR "trial s"[All Fields] OR "trialed"[All Fields] OR "trialing"[All Fields] OR "trials"[All Fields]) OR "clinical trial"[All Fields])	All Fields	147
WOS	(("aldosterone synthase inhibitor" OR "aldosterone synthase inhibitors" OR "aldosterone synthase" OR "CYP11B2 inhibitor" OR "CYP11B2 inhibitors" OR "aldosterone synthase inhibitor*" OR "CYP11B2") AND (hypertens* OR "resistant hypertens*" OR "refractory hypertens*" OR "treatment-resistant hypertens*" OR "drug-resistant hypertens*" OR "high blood pressure") AND (Random* OR "randomized controlled triaL" OR randomized OR randomised OR randomly OR trial OR "clinical trial"))	All Fields	165
SCOPUS	TITLE-ABS-KEY (("aldosterone synthase inhibitor" OR "aldosterone synthase inhibitors" OR "aldosterone synthase" OR "CYP11B2 inhibitor" OR "CYP11B2 inhibitors" OR "aldosterone synthase inhibitor*" OR "CYP11B2") AND (hypertens* OR "resistant hypertens*" OR "refractory hypertens*" OR "treatment-resistant hypertens*" OR "drug-resistant hypertens*" OR "high blood pressure") AND (Random* OR "randomized controlled triaL" OR randomized OR randomised OR randomly OR trial OR "clinical trial"))	Title, Abstract, Keywords	273
Cochrane	(("aldosterone synthase inhibitor" OR "aldosterone synthase inhibitors" OR "aldosterone synthase" OR "CYP11B2 inhibitor" OR "CYP11B2 inhibitors" OR "aldosterone synthase inhibitor*" OR "CYP11B2") AND (hypertens* OR "resistant hypertens*" OR "refractory hypertens*" OR "treatment-resistant hypertens*" OR "drug-resistant hypertens*" OR "high blood pressure") AND (Random* OR "randomized controlled triaL" OR randomized OR randomised OR randomly OR trial OR "clinical trial"))	All Fields	53
